# Assessment of l-Asparaginase Pharmacodynamics in Mouse Models of Cancer

**DOI:** 10.3390/metabo9010010

**Published:** 2019-01-09

**Authors:** Thomas D. Horvath, Wai Kin Chan, Michael A. Pontikos, Leona A. Martin, Di Du, Lin Tan, Marina Konopleva, John N. Weinstein, Philip L. Lorenzi

**Affiliations:** 1 Department of Bioinformatics and Computational Biology and The Proteomics and Metabolomics Core Facility, The University of Texas MD Anderson Cancer Center, Houston, TX 77030, USA; thomasdhorvath@gmail.com (T.D.H.); wkchan@mdanderson.org (W.K.C.); mapontikos@gmail.com (M.A.P.); lamartin1@mdanderson.org (L.A.M.); dudthu06@gmail.com (D.D.); ltan@mdanderson.org (L.T.); jweinste@mdanderson.org (J.N.W.); 2 Department of Leukemia, The University of Texas MD Anderson Cancer Center, Houston, TX 77030, USA; mkonople@mdanderson.org; 3 Department of Stem Cell Transplantation, The University of Texas MD Anderson Cancer Center, Houston, TX 77030, USA

**Keywords:** Kidrolase, Erwinaze, asparaginase, glutaminase, pharmacodynamics, targeted metabolomics

## Abstract

l-asparaginase (ASNase) is a metabolism-targeted anti-neoplastic agent used to treat acute lymphoblastic leukemia (ALL). ASNase’s anticancer activity results from the enzymatic depletion of asparagine (Asn) and glutamine (Gln), which are converted to aspartic acid (Asp) and glutamic acid (Glu), respectively, in the blood. Unfortunately, accurate assessment of the in vivo pharmacodynamics (PD) of ASNase is challenging because of the following reasons: (i) ASNase is resilient to deactivation; (ii) ASNase catalytic efficiency is very high; and (iii) the PD markers Asn and Gln are depleted ex vivo in blood samples containing ASNase. To address those issues and facilitate longitudinal studies in individual mice for ASNase PD studies, we present here a new LC-MS/MS bioanalytical method that incorporates rapid quenching of ASNase for measurement of Asn, Asp, Gln, and Glu in just 10 µL of whole blood, with limits of detection (s:n ≥ 10:1) estimated to be 2.3, 3.5, 0.8, and 0.5 µM, respectively. We tested the suitability of the method in a 5-day, longitudinal PD study in mice and found the method to be simple to perform with sufficient accuracy and precision for whole blood measurements. Overall, the method increases the density of data that can be acquired from a single animal and will facilitate optimization of novel ASNase treatment regimens and/or the development of new ASNase variants with desired kinetic properties.

## 1. Introduction

l-Asparaginase (ASNase; EC 3.5.1.1) is an amidohydrolase enzyme that catalytically deamidates l-asparagine (Asn) to l-aspartic acid (Asp) and ammonia, and, to a lesser degree, l-glutamine (Gln) to l-glutamic acid (Glu) and ammonia. After early reports of the anti-lymphoma activity possessed by guinea pig serum [1,2], ASNase was determined to be responsible for the activity [3]. Currently, only the variants from *Escherichia coli* (Medac^®^ (Medac GmbH, Wedel, Germany), Kidrolase^®^ (Jazz Pharmaceuticals, Dublin, Ireland), and Spectrila^®^ (Medac GmbH, Wedel, Germany), and the pegylated enzyme, Oncaspar^®^ (Takeda Pharmaceuticals, Osaka, Japan)) and *Erwinia chrysanthemi* (Erwinaze^®^ (Jazz Pharmaceuticals, Dublin, Ireland)) have been approved for the treatment of cancer. Other forms have been tested but were found to be too toxic; for example, clinical trials with *Wolinella succinogenes* ASNase were terminated due to toxicity. It is generally thought that ASNase-mediated depletion of Asn in the blood plasma is an effective therapy for cancer cells that express asparagine synthetase (ASNS; EC 6.3.5.4) at low levels and, hence, depend on systemic Asn to support their growth and proliferation. In fact, a causal association between ASNase anticancer activity and ASNS expression has been demonstrated [4,5,6,7,8]. Asn-starved leukemia cells exhibit a global decrease in protein biosynthesis that ultimately results in cell death [9,10,11,12,13].

**Figure 1:**
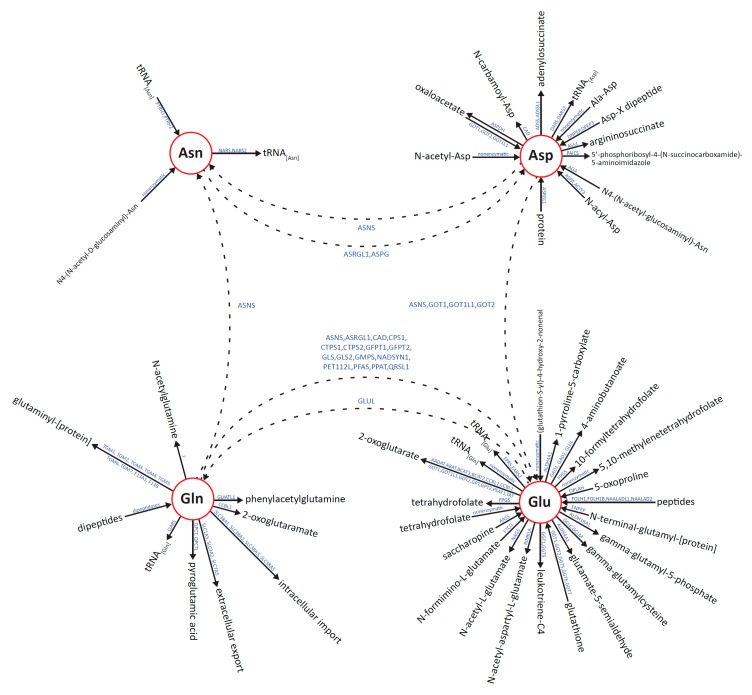
“Metaburst” of metabolic pathways modulated by ASNase, including biological reactions associated with the metabolites asparagine (Asn), aspartic acid (Asp), glutamine (Gln), and glutamic acid (Glu). All reactions are also listed in Table S1.

Targeting metabolism is a prominent strategy in the treatment of cancer, and ASNase targets a key set of metabolic pathways centered on its targets Asn and Gln, which affect a wide range of downstream metabolites, as shown in Figure 1 and Table S1. Despite ongoing efforts to optimize the enzyme’s ratio of asparaginase:glutaminase activity, numerous challenges persist with regard to optimizing clinical outcomes with ASNase therapy. One significant issue is that therapeutic drug monitoring of plasma ASNase activity must be conducted to ensure that Asn levels are effectively depleted [14]. 

Unfortunately, technical challenges have hindered adoption of therapeutic drug monitoring methods. One challenge stems from the resilience of the enzyme to quenching [15,16]. A second challenge is its high catalytic efficiency (k_cat_/K_m_ approximately 1 × 10^6^ M^−1^s^−1^) [17]. Consequently, even at the relatively low concentration of 0.1 IU/mL, ASNase fully depletes physiological concentrations of Asn within seconds [18]. Third, the pharmacodynamic (PD) markers Asn and Gln are depleted ex vivo in blood samples from patients treated with ASNase, thereby introducing analytical artifacts. A method that successfully quenches ASNase activity immediately upon blood collection by the addition of sulfosalicylic acid (SSA) has been reported [16] but requires large blood volumes (greater than 2 mL) and derivatization of the amino acids prior to chromatographic separation and fluorescence detection. Herein, we describe a liquid chromatography-tandem mass spectrometry (LC-MS/MS)-based bioanalytical method that rapidly quenches ASNase activity, demonstrates acceptable precision and accuracy across the normal range (NR) of Asn, Asp, Gln, and Glu that are typical in mouse whole blood, and has sufficient sensitivity to limit the sample volume to 10 µL, facilitating longitudinal studies in individual mice that have been treated with ASNase.

## 2. Results

### 2.1. Optimization of Amino Acid Acquisition Parameters and ASNase Activity Quenching

We first optimized the acquisition parameters on an Agilent 6460 triple quadrupole mass spectrometer using Agilent Optimizer Software (Version B.06.00) and post-column infusion; molecule-specific acquisition parameters for the analytes and internal standards are described in Table 1.

**Figure 2:**
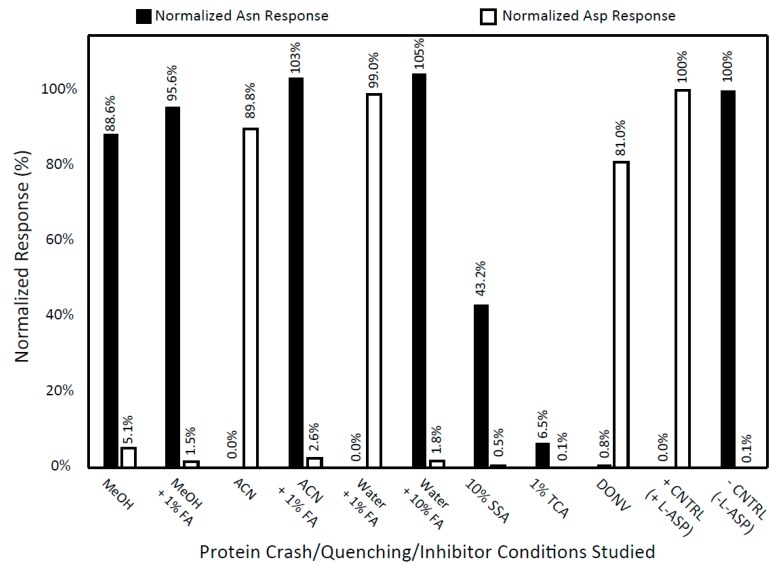
Screen for ASNase Activity Quenchers. The following conditions were tested for the ability to quench the conversion of Asn to Asp by ASNase: (1) 80:20 methanol (MeOH):water; (2) 80:20 MeOH containing 1% formic acid (FA):water; (3) 80:20 acetonitrile (ACN):water; (4) 80:20 ACN containing 1% FA:water; (5) 80:20 water containing 1% FA:water; (6) 80:20 water containing 10% FA:water; (7) 80:20 water containing 10% (*w*/*v*) sulfosalicylic acid (SSA):water; (8) 80:20 water containing 1% (*w*/*v*) trichloroacetic acid (TCA):water; (9) 80:20 water containing 40 mM 5-diazo-4-oxo-l-norvaline (DONV):water; (10) positive control sample containing ASNase in water; and (11) negative control sample containing water without ASNase. Data are presented as normalized response of Asn and Asp. The final activity of ASNase and solution concentration of Asn in each sample tested was approximately 20 IU/mL and 100 µM, respectively.

Since quenching of ASNase is a key prerequisite for the accurate measurement of Asn, Asp, Gln, and Glu in the presence of ASNase, we first screened a range of organic solvents and organic acids for the ability to neutralize ASNase enzyme activity. The results illustrated in Figure 2 clearly show that methanol was found to be superior to acetonitrile in terms of ASNase quenching; Asn was almost completely converted to Asp in 20:80 water:acetonitrile. Given that 80% acetonitrile is widely used for the precipitation of protein from biological samples, our observations underscore the resilience of the ASNase enzyme to quenching. Another unexpected result was our observation of a chromatography issue for Asn by the presence of SSA in neat samples as shown in Supplementary Figure S1, which was shown previously to be an effective quencher of ASNase for a published LC-fluorescence based bioanalytical method [16]. Additional method development for an alternate extraction method (e.g., solid phase extraction) that removes SSA from the sample extract may eliminate the chromatography issue, but since we found alternative, effective ASNase quenching conditions that are compatible with our chromatographic system, we have not explored the use of SSA further. ASNase was successfully quenched by: (i) water containing 10% formic acid (FA), (ii) methanol containing 1% FA, and iii) acetonitrile containing 1% FA. Hence, we have chosen to incorporate those solvents into the method as the quencher, the protein precipitation solvent, and a component of the reconstitution solvent, respectively.

### 2.2. Accuracy, Precision, Recovery, Normalized Matrix Factor, and FTS Assessments

Analytical figures of merit were assessed through the preparation, extraction, and analysis of five analytical batches, each containing six replicates at each quality control (QC) level (*n* = 30 replicates overall for each level) over five non-sequential days. Inter-day precision and accuracy at each QC level were defined as the coefficient of variation (%CV; standard deviation divided by the mean multiplied by 100) and percent relative error (%RE = (([AA]_mean_/[AA]_nominal_)-1) * 100), respectively. The resulting precision and accuracy data for the three QC levels studied are provided in Table 2. The accuracy of the mean concentrations for Gln and Asn were within 15% for all QC levels studied. The accuracy of the QC-Mid and QC-High levels for Glu and Asp were within 15%, but the accuracy of the QC-Low level in both instances was equal to or greater than 20%, which indicates that the method for these two analytes may lack the precision at the low end to discriminate between the dialyzed whole blood (DWB) matrix background and the exogenous levels of Asp and Glu contained in the QC-Low sample. Ultimately, because those two analytes are products of the ASNase reaction, the observed decrease in accuracy (increase in %RE) at QC-Low should not pose significant problems for the assay, since the in vivo whole blood concentration of Glu and Asp in the presence of ASNase are expected to increase over their empirically determined NR (79–122 µM for Glu and 30–47 µM for Asp in mouse whole blood). The background concentrations of Asn, Asp, Gln, and Glu remaining in the DWB matrix after the dialysis procedure (described in the Materials and Methods Section) were (fold-change below NR indicated in parentheses) 0.94 µM (46-fold), 1.49 µM (19-fold), 1.65 µM (348-fold), and 3.62 µM (24-fold), respectively. The limit of detection for Asn, Asp, Gln, and Glu in just 10 µL of whole blood were estimated to be 2.3, 3.5, 0.8, and 0.5 µM, respectively.

The normalized matrix factor (NMF) was calculated using Equation (1):

(1) $$NMF=\left(\frac{\left(\frac{Area_{analyte,\;post}}{Area_{Analyte,\;neat}}\right)}{\left(\frac{Area_{IS,\;post}}{Area_{IS,\;neat}}\right)}\right)$$

where *Area_analyte, post_* and *Area_IS, post_*, and *Area_analyte, neat_* and *Area_IS, neat_* are analyte and internal standard (IS) peak areas from the post-extraction DWB sample matrix and neat samples (a water blank is extracted and dried, and analyte and IS are added during the sample reconstitution step), respectively. The results for NMF and recovery assessments for each analyte and IS are provided in Table 3. Gln and Gln-IS exhibited the lowest mean recoveries at around 90% for the three levels studied, but all of the other analytes and IS compounds had recoveries near 100%. The NMF was approximately 1.0 for all analyte and IS compounds at all concentration levels tested, indicating that the degree of ion enhancement or suppression effects between each analyte/IS pair in the DWB matrix was equivalent.

### 2.3. Pharmacodynamics of ASNase in NOD.Cg-PRKDC(scid) IL2RG(tm1Wjl) (NSG) Mice

**Figure 3:**
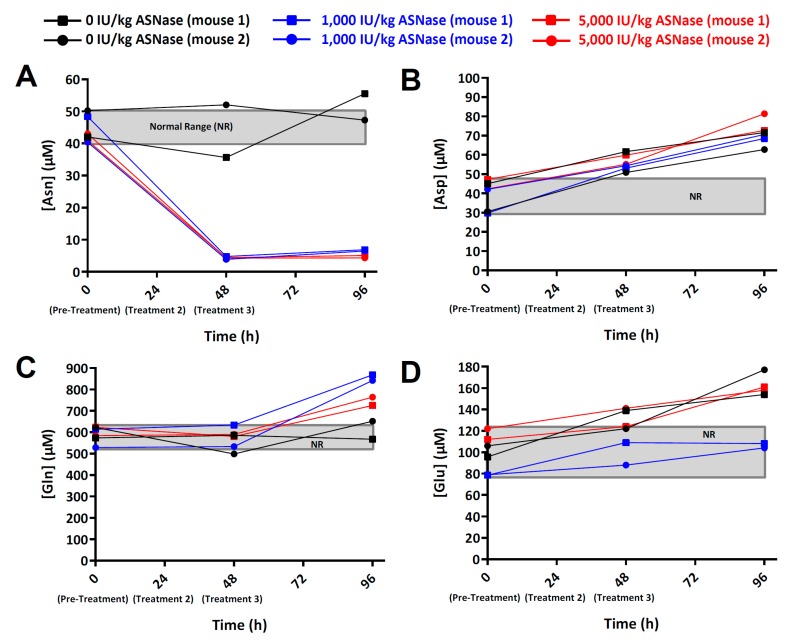
Amino acid concentration (µM) versus time (h) in whole blood of mice treated with ASNase. (**A**) asparagine (Asn), (**B**) aspartic acid (ASP), (**C**) glutamine (Gln), and (**D**) glutamic acid (Glu). Each data series represents an individual mouse, and the mice were arranged into the following three cohorts that were given intraperitoneal injections of either vehicle (1x PBS) or ASNase at 0, 24, and 48 h: (1) Control mice (*n* = 2; blackline with black squares and circles); (2) mice treated with 1000 IU/(kg·day) ASNase (*n* = 2; blue lines with blue squares and circles); (3) mice treated with 5000 IU/(kg·day) ASNase (*n* = 2red lines with red squares and circles). ASNase was administered at 0, 24, and 48 h, and whole blood (10 µL) was collected from each mouse at 0, 48, and 96 h, and was processed as described. The gray box represents the NR levels of Asn, Asp, Gln, and Glu defined by the 0 h (pre-treatment) samples.

We conducted a pilot study in three treatment groups of NSG mice (control, low-dose ASNase, and high-dose ASNase) to determine the suitability of the bioanalytical method for assessing ASNase PD in the mouse model of leukemia. The NR was defined as the largest absolute concentration range measured for each analyte, and the NR for each metabolite was: Asn: 40–50 µM; Asp: 30–47 µM; Gln: 528–623 µM; Glu: 79–122 µM as shown in Figure 3. Note: The connecting lines for each data series are used to visually connect the data points within each individual mouse; analyte concentrations should not be inferred from the lines between adjacent data points.

The ASNase field has historically used plasma as a biological matrix for ASNase PD. Our use of whole blood offers the notable advantage of rapid quenching of ASNase, but a weakness is that red blood cell (RBC) volume can be variable. However, since individual subjects are expected to have low variability in RBC volume, our combined use of whole blood and longitudinal methodology minimizes the variability associated with hematocrit. Although the pilot study was originally designed as a simple test case to assess the method performance, several interesting biological observations were made that warrant further studies. First, we found that Asn blood levels were detectable following treatment with ASNase, whereas previously published methods invariably yielded post-ASNase Asn levels “below the limit of detection/quantitation” [19,20,21,22]. The improved detection of Asn with our new method is partially due to the fact that we used whole blood, which captures target analytes in both the red blood cell (RBC) and plasma compartments, whereas other methods typically use serum or plasma. From the perspective of ASNase neutralization/quenching, whole blood sampling may yield more accurate results, since whole blood can be quickly quenched after collection to eliminate artifactual ex vivo depletion of Asn, whereas plasma and serum require additional time (between 3 and 10 min of centrifugation time for whole blood to plasma processing) during which even low levels of ASNase activity are able to deplete large quantities of Asn. Second, although previously published methods [23,24,25] report lower Limit of Detection (LOD) and Limit of Quantitation (LOQ) levels, those methods typically require blood volumes that range from 200 µL to 2 mL—volumes that are not compatible with longitudinal studies in individual mice (total blood volume in an individual mouse is ~1 mL). The precision and accuracy obtained from just 10 µL of whole blood now make it possible to conduct longitudinal studies of individual mice. Third, the results suggest that the method is suitable for measuring repletion of Asn after ASNase cessation as shown in Figure 3A; mean Asn concentrations at 96 h (48 h after the final L-ASP dose) for the 1,000 IU/(kg·day) and the 5,000 IU/(kg·day) dosages were 6.66 µM (*n* = 2; blue lines with blue squares and circles) and 4.72 µM (*n* = 2; red lines with red squares and circles), respectively. Overall, these biological observations suggest that new investigations should be undertaken to interrogate features of the biological response to ASNase treatment, including efforts to identify the true whole blood concentration of Asn that is thought to trigger cancer cell death.

Blood levels of Asp tended to increase above the NR for all animals (including the control mice) by the 48 h timepoint as shown in Figure 3B. Since the disappearance of Asn was not matched by a commensurate (stoichiometric) appearance of Asp within the ASNase-treated groups, the results prompt the hypothesis that Asp is tightly regulated with excess amounts being shunted to other metabolite pools/pathways. Candidate pathways include: (1) ASNS-mediated conversion of Asp to Asn with a corresponding conversion of Gln to Glu; and (2) aspartate aminotransferase (AST/GOT1/GOT2; EC 2.6.1.1)-mediated conversion of Asp to Glu with a corresponding conversion of alpha-ketoglutaric acid to oxaloacetic acid as show in Figure 1. Further work to discern those possibilities is warranted.

The blood level of Gln at the 48 h time point (24 h after the second dose of ASNase) appeared to be near the NR defined by the 0 h time points, but at 96 h Gln levels exhibited dose-dependent up-regulation, with the 1,000 IU/kg ASNase dose yielding a larger up-regulation of Gln than the 5,000 IU/kg ASNase dose as shown in Figure 3C. The corresponding levels of Glu, by contrast, did not differ commensurately with Gln, and that was also the case for Asp, which was not modulated commensurately with Asn. One potential explanation for those results is that glutamine synthetase (GLUL; EC 6.3.1.2), which catalyzes the condensation of Glu and ammonia to produce Gln, is up-regulated at the tissue-level. Indeed GLUL has been reported to be up-regulated by ASNase treatment [26]. A number of additional enzymes that may rapidly metabolize ASNase-generated Glu to maintain steady-state levels are listed in Figure 1.

## 3. Discussion

Here we present a bioanalytical method that enables the direct measurement of the ASNase PD markers Asn, Asp, Gln, and Glu by LC-MS/MS without sample derivatization, building upon previously reported methods [27]. The method is sufficiently sensitive to measure the PD markers in as little as 10 µL of whole blood, thus facilitating longitudinal studies in individual small animals such as mice. Moreover, the method uses experimental conditions (e.g., water containing 10% FA for ASNase quenching, methanol containing 1% FA for protein precipitation, and acetonitrile containing 1% FA as a component of the reconstitution solution) that we have shown to be effective at quenching ASNase activity, thereby eliminating ex vivo turnover of Asn and Gln in the processed sample extracts prior to analysis. Application of the method to a small pilot study that included three cohorts of mice treated with increasing doses of ASNase yielded interesting biological observations that warrant further study: (i) ASNase treatment did not appear to modulate blood levels of Asp, contrary to our expectation of a stoichiometric increase in Asp concentration commensurate with ASNase-mediated depletion of Asn; (ii) unexpectedly large increases of Gln after cessation of ASNase treatment prompt the hypothesis that glutamine synthetase (GLUL) is up-regulated at a systems-level (perhaps in specific organs) in response to ASNase treatment. Overall, the method promises to improve our understanding of the mechanisms that mediate sensitivity and resistance to ASNase.

## 4. Materials and Methods

### 4.1. Reagents and Chemicals

Optima™ LC-MS-grade acetonitrile, methanol, and water, and a 1M hydrochloric acid solution were purchased from Thermo Fisher Scientific (Waltham, MA, USA). Formic acid (98%), ammonium formate, SSA, and trichloroacetic acid (TCA) were purchased from Sigma-Aldrich (St. Louis, MO, USA). Authentic reference materials and stable-isotope labeled internal standards including Asn, [^13^C_4_,^15^N_2_]-Asn, Asp, [^13^C_4_,^15^N_1_]-Asp, Gln, [^13^C_5_,^15^N_2_]-Gln, Glu, and [^13^C_5_,^15^N_1_]-Glu were purchased from Sigma-Aldrich. Kidrolase^®^ (ASNase) was purchased from Jazz Pharmaceuticals. The l-Asn analog and irreversible ASNase inhibitor, 5-diazo-4-oxo-l-norvaline (DONV), was synthesized by Acme Bioscience (Palo Alto, CA, USA).

### 4.2. Equipment and Consumables

In this study, 1.4 mL polypropylene Matrix tubes and SepraSeal caps were purchased from Thermo Fisher Scientific and were used for frozen storage of dialyzed whole blood (DWB) matrix, DWB-derived quality control (QC) standards, and for the extraction of all study samples. Micrewtube^®^ tubes (2.0 mL) and caps were purchased from Simport (Montreal, QC, Canada) and were used for frozen storage of the calibration (calibrator) standard spiking solutions. Slide-A-Lyzer Dialysis Cassette G2 (10,000 MWCO; 15 mL capacity) from Thermo Fisher Scientific was used to dialyze lysed whole blood matrix. A Kinetex HILIC (100 × 2.1 mm, 1.7 µm particle size) analytical column from Phenomenex (Torrance, CA, USA) was used for chromatographic separation of ASNase PD markers. Chromatographic data were acquired using a 1290 Infinity Binary UHPLC system coupled to a 6460 tandem-mass spectrometer produced by Agilent Technologies (Santa Clara, CA, USA). MassHunter LC/MS Data Acquisition Software (Version B.06.00) was used for control and operation of the LC-MS/MS system, and MassHunter Quantitative Analysis Software (Version B.06.00) was used for chromatographic peak integration. Weighted (1/x) linear regression analysis for each calibration curve was performed using GraphPad Prism (Version 6.05) from GraphPad Software (La Jolla, CA, USA).

### 4.3. Optimization of Experimental Conditions for the Effective Quenching of ASNase Activity

Overall, nine different experimental conditions were tested for ASNase quenching efficacy in freshly prepared neat solutions (i.e., fresh solvent mixtures, or solvents containing acids or inhibitors in the absence of biological material). We tested the following solutions: (1) four organic solvent solutions with and without formic acid (methanol, methanol with 1% formic acid, acetonitrile, and acetonitrile with 1% formic acid); (2) two aqueous solutions that consisted of water with 1% formic acid, and water with 10% formic acid; (3) two aqueous solutions of protein precipitation reagents that consisted of water with 10% (*w*/*v*) SSA and water with 1% (*w*/*v*) TCA; and (4) one aqueous solution that consisted of water with 40 mM DONV. A non-quenched positive control sample (+ASNase) and a negative control sample lacking ASNase (-ASNase) were also prepared with a consistent Asn concentration for normalization purposes. All of the solutions, samples, and controls were prepared fresh on the day of the experiment and tested as a single replicate in a single batch.

The quenching study samples were prepared by adding 100 µL of an ASNase spiking solution (100 IU/mL) prepared in water, and 400 µL of the appropriate quenching test solution to a labeled sample tube, and all of the samples were briefly vortex-mixed. Next, 10 µL of an Asn spiking solution (5000 µM) solution was added to each sample, and each sample was vortex-mixed briefly. The samples were allowed to incubate at room temperature for 10 min, then they were centrifuged at 17,000× *g* for 5 min at 4 °C. The supernatant was transferred to polypropylene autosampler vials and analyzed on the LC-MS/MS system. The final activity of ASNase and solution concentration of Asn in each sample tested was approximately 20 IU/mL and 100 µM, respectively.

### 4.4. Dialysis of Mouse Whole Blood

A mixed sex, pooled lot of K2-EDTA CD-1 mouse whole blood was purchased from BioreclamationIVT (New York, NY, USA). Upon receipt, the whole blood matrix was frozen at −20 °C for a minimum of 24-h to ensure complete RBC lysis. Prior to dialysis, the whole blood matrix was thawed, then centrifuged at 4500× *g* for 10 min to pellet the RBC debris, and finally the whole blood supernatant was dialyzed (10,000 MWCO membrane) against 5 L of 1× PBS over five 24-h passages at +4 °C. The DWB matrix was then transferred to polypropylene tubes and stored at −20 °C until needed. The mouse DWB matrix was used in the preparation of calibrators and QCs. The endogenous background levels of Asn, Asp, Gln, and Glu that remained in the DWB matrix after dialysis were determined by triplicate analysis of blank samples in each analytical run.

### 4.5. Preparation of Amino Acid Stock, Combined Intermediate, and Calibrator Spiking Solutions

Individual Asn and Gln stock solutions were prepared in a solution of water that contained 1% formic acid. Individual Asp and Glu stock solutions were prepared in a solution of water that contained 1 M hydrochloric acid to ensure solubility. The nominal concentration for each stock solution was corrected to account for salt form, purity, and water content reported on the reference material product literature. Individual stock solutions were used to prepare a combined intermediate solution that contained each analyte component at a concentration of 10,000 µM each. The combined intermediate solution was serially diluted further to prepare calibrator spiking solutions at the following concentrations: 4.00, 8.00, 100, 400, 1000, 2000, 3600, and 4000 µM. All dilutions were made using a solution of water that contained 1% formic acid as the diluent. The individual stock solutions, combined intermediate, and the individual calibrator spiking solutions were stored in polypropylene tubes and at −80 °C when not in use.

### 4.6. Preparation of Stable-Isotope Labeled Internal Standard Stock and Working Internal Standard Solutions

Individual [^13^C_4_,^15^N_2_]-Asn and [^13^C_5_,^15^N_2_]-Gln stock solutions were prepared in a solution of water that contained 1% formic acid. Individual [^13^C_4_,^15^N_1_]-Asp and [^13^C_5_,^15^N_1_]-Glu stock solutions were prepared in a solution of water that contained 1 M hydrochloric acid to ensure solubility. The nominal concentration for each internal standard stock solution was corrected to account for salt form, purity, and water content reported on the reference material product literature. The individual internal standard stock solutions were used to prepare a working internal standard (WIS) solution that contained each internal standard component at a concentration of 1,000 µM each. All dilutions were made using a solution of water that contained 1% formic acid as the diluent. The individual internal standard stock and WIS solutions were stored in polypropylene tubes and at −80 °C when not in use.

### 4.7. Sample Extraction Procedure

The following sample extraction procedures were performed for all analytical batch preparations described in this study. All samples immediately received a 30 µL aliquot of water that contained 20% formic acid, which was added to quench ASNase activity in the study samples. All samples were capped and vortex-mixed for 30 s. All samples received a 240 µL aliquot of a solution of methanol that contained 1% formic acid to precipitate protein. All samples were vortex-mixed for two min and centrifuged for ten min at 17,000× *g* at ambient temperature. Supernatant was then transferred to a new sample tube and evaporated to dryness in the Savant vacuum concentrator with sample heating at 45 °C. The collection and extraction procedures used for the ASNase PD study samples is described below.

### 4.8. Sample Reconstitution

Samples were reconstituted according to the following two-step process: (1) Each sample received a 20 µL aliquot of a solution of water that contained 1% formic acid, was capped, and was vortex-mixed for 30 s to solubilize glutamic acid and aspartic acid prior to the addition of organic solvent; (2) Each sample then received a 180 µL aliquot of a solution of acetonitrile that contained 1% formic acid, was capped, and was vortex-mixed for an additional 2 min. All samples were again centrifuged at 17,000× *g* for ten min, and the supernatant was transferred to polypropylene injection vials (Thermo Fisher Scientific). The sample extracts were stored in a refrigerator until analysis, and a 5 µL sample volume was injected onto the instrument for analysis.

### 4.9. Liquid Chromatography/Mass Spectrometry Conditions

Hydrophilic Interaction Chromatography (HILIC) mobile phase A (MPA; weak) and mobile phase B (MPB; strong) solutions used for this study were acetonitrile/200 mM ammonium formate/formic acid (950:50:20; *v*:*v*:*v*), and acetonitrile/water/200 mM ammonium formate/formic acid (500:450:50:20; *v*:*v*:*v*:*v*), respectively. The chromatographic column was a Phenomenex Kinetex HILIC analytical column (2.1 × 100 mm, 1.7 µm particle size). The chromatographic method included column heating at 30 °C, autosampler tray chilling at +4 °C, a mobile phase flowrate of 0.200 mL/min, and a gradient elution program specified as follows: 0–2.5 min, 5% MPB; 2.5–10.5 min, 5–90% MPB; 10.5–12 min, 90–5% MPB; 12–14 min, 5% MPB. The overall cycle-time for a single injection was approximately 14.5 min. Representative extracted-ion chromatograms for the analytes and internal standards can be found in Supplementary Figure S2.

The Agilent Jet Stream-electrospray ionization (AJS-ESI) source was installed on the mass spectrometer and operated in unit/unit resolution and positive ionization mode with the following acquisition parameters: gas temperature: 325 °C; gas flow: 6 L/min; nebulizer: 40 psi; sheath gas temperature: 350 °C; sheath gas flow: 9 L/min; capillary voltage: +1250 V; nozzle voltage: +500 V. All reference and internal standard compounds were optimized using the Agilent Optimizer Software (Version B.06.00) and post-column infusion; molecule-specific acquisition parameters for the analytes and internal standards are described in Table 1.

### 4.10. Study Design

This study was performed in a pathogen-free vivarium at The University of Texas MD Anderson Cancer Center under an approved Institutional Animal Care and Use Committee (IACUC) study protocol (ACUF #00001658-RN00). The study included five NOD.Cg-PRKDC(scid) IL2RG(tm1Wjl) (NSG; stock #005557) mice purchased from The Jackson Laboratory (Bar Harbor, ME). The five mice were arranged into three treatment groups: (1) control (*n* = 2; 100 µL PBS administered by intraperitoneal (IP) injection every day for three days); (2) low-dose (*n* = 2; 100 µL PBS containing 1000 IU Kidrolase per kg body weight administered IP every day for three days); (3) high-dose (*n* = 2; 100 µL PBS containing 5000 IU Kidrolase per kg body weight administered IP every day for three days). Each treatment was administered at 0, 24, and 48 h after study initiation. Whole blood (10 µL) was collected from each mouse before study initiation (pre-dose) and again at 48 and 96 h (48 h after cessation of treatment). When whole blood was collected on treatment days, it was collected immediately before the administration of the treatment.

### 4.11. Preparation of Sample Extraction Tubes

Prior to blood collection, the following solutions were added to individual extraction tubes corresponding to each sample: (1) 10 µL of WIS solution; (2) 10 µL of a solution of water that contained 1% formic acid to make up for the spiking solution volume in the preparation of the Calibrators; and (3) 30 µL of a solution of water that contained 20% formic acid to quench the enzymatic activity of ASNase present in the whole blood samples. Finally, all sample tubes were vortex-mixed for approximately 30 s, centrifuged at 3,000× *g* for approximately 1 min, appropriately labeled for a specific sample, and stored on ice for transport to the vivarium.

### 4.12. Collection, ASNase Activity Quenching, and Extraction of Mouse Whole Blood Study Samples

Using a sterile tail-snip method, a 10 µL whole blood sample was collected from the tail of each mouse and was immediately transferred to the appropriately labeled extraction tube containing the stable isotope-labeled IS compounds. Each sample was briefly vortexed to thoroughly mix the whole blood with the contents of the extraction tube. Complete RBC lysis and protein precipitation was ensured by the addition of 240 µL of an ice-cold solution of methanol that contained 1% formic acid. Samples were stored on ice, transported back to the laboratory, vortex-mixed for two min, and centrifuged for ten min at 17,000× *g* at ambient temperature. Supernatant was then transferred to a new sample tube and evaporated to dryness in a Savant vacuum concentrator with sample heating at 45 °C. Dried sample extracts were capped and stored at −80 °C until reconstitution.

### 4.13. Quantitative Analysis Workflow

A custom quantitative analysis workflow had been devised to correct for the detectable levels of Asn, Asp, Gln, and Glu contained in the DWB matrix that was used to prepare the calibrator samples. Chromatographic peak integrations were performed using the MassHunter Quantitative Analysis software package. When peak integrations were completed for each individual analyte, the results table was exported into a custom Excel spreadsheet that was designed to automate the following computational tasks: (1) compute the mean area response for the analyte contained in the blank whole blood matrix; (2) subtract the mean analyte response in the whole blood matrix from the analyte response measured for each calibrator level; and (3) compute a corrected instrument response (IR ≡ [Corrected Area]_analyte_/[Area]_IS_) factor for each calibrator level. Individual calibration curves were generated in GraphPad Prism by performing a least-squares linear regression with 1/x weighting on plots of IR factors versus nominal analyte concentration for each calibrator. Individual slope and intercept outputs from the linear regression analysis for each calibration curve were input into the spreadsheet in order to compute the analyte concentration of each sample present in that batch.

## Figures and Tables

**Table 1:** Molecule-specific MS/MS parameters.

Compound	SRM^a^ (*m*/*z*)	Fragmentor Voltage (V)	Collision Energy (V)
[^13^C_0_]-Asn	133.1 → 74.1	45	17
[^13^C_4_,^15^N_2_]-Asn	133.1 → 74.1	45	17
[^13^C_0_]-Asp	134.0 → 74.1	45	13
[^13^C_4_,^15^N_1_]-Asp	139.1 → 77.1	45	13
[^13^C_0_]-Gln	147.1 → 84.1	45	5
[^13^C_4_,^15^N_2_]-Gln	154.1 → 89.1	45	5
[^13^C_0_]-Glu	148.1 → 84.1	45	17
[^13^C_4_,^15^N_2_]-Glu	154.1 → 89.1	45	17

^a^ Selected reaction monitoring.

**Table 2:** Inter-day mean concentration, accuracy, and precision for QC standards prepared in dialyzed whole blood matrix.

[Asn]_nominal_ (µM)	[Asn]_mean_ (µM) ^a^	Accuracy (%RE) ^b^	Precision (%CV) ^c^
12.0	10.6	−11.8%	4.78%
200	182	−9.00%	2.31%
3200	3140	−1.89%	2.38%
[Asp]_nominal_ (µM)	[Asp]_mean_ (µM) ^a^	Accuracy (%RE) ^b^	Precision (%CV) ^c^
12.0	14.6	21.9%	7.05%
200	214	7.16%	3.75%
3200	3155	1.41%	2.57%
[Gln]_nominal_ (µM)	[Gln]_mean_ (µM) ^a^	Accuracy (%RE) ^b^	Precision (%CV) ^c^
12.0	11.1	−7.87%	5.77%
200	215	7.60%	2.32%
3200	3675	14.8%	2.50%
[Glu]_nominal_ (µM)	[Glu]_mean_ (µM) ^a^	Accuracy (%RE) ^b^	Precision (%CV) ^c^
12.0	14.4	20.0%	6.13%
200	209	4.36%	2.16%
3200	3122	−2.42%	1.56%

^a^ The mean concentration was calculated from 1/x weighted linear least-squares regressions from the individual calibration curves in each batch (*n* = 5 over five non-consecutive days) after correcting for the endogenous amino acid content contained in the dialyzed whole blood matrix; ^b^ percent relative error: %RE = (([AA]_mean_/[AA]_nominal_)-1) * 100; ^c^ coefficient of variation.

**Table 3:** Mean recovery and mean normalized matrix factors (NMF) for three different quality control levels.

[Analyte]/[IS] ^a^	Mean Recovery Asn/Asn-IS ^b^	Mean Recovery Asp/Asp-IS ^c^	Mean Recovery Gln/Gln-IS ^d^	Mean Recovery Glu/Glu-IS ^e^
8.00 µM/100 µM	94%/98%	97%/97%	87%/89%	96%/99%
1000 µM/100 µM	96%/96%	101%/99%	91%/92%	100%/100%
4000 µM/100 µM	98%/98%	100%/98%	92%/92%	99%/99%
[Analyte]/[IS] ^a^	Mean NMF Asn/Asn-IS ^b^	Mean NMF Asp/Asp-IS ^c^	Mean NMF Gln/Gln-IS ^d^	Mean NMF Glu/Glu-IS ^e^
8.00 µM/100 µM	0.971	0.966	0.956	0.967
1000 µM/100 µM	1.00	0.972	0.996	0.999
4000 µM/100 µM	0.998	1.01	1.00	0.993

^a^ IS: internal standard; ^b^ Asn-IS: [^13^C_4_,^15^N_2_]-asparagine; ^c^ Asp-IS: [^13^C_4_,^15^N_1_]-aspartic acid; ^d^ Gln-IS: [^13^C_5_,^15^N_2_]-glutamine; ^e^ Glu-IS: [^13^C_5_,^15^N_1_]-glutamic acid.

## Supplementary Materials

The following are available online at http://www.mdpi.com/2218-1989/9/1/10/s1, Figure S1: Sulfosalicylic acid exhibits an effect on Asn chromatography; Figure S2: Extracted-ion chromatograms (XIC) for the transitions monitored; Table S1: Compiled biological reactions involving asparagine, aspartic acid, glutamine, and glutamic acid.
