# Supplementary material for: Assessment of l-Asparaginase Pharmacodynamics in Mouse Models of Cancer
Source: Metabolites. 2019 Jan 9;9(1):10. doi: 10.3390/metabo9010010 (PMC6359345; doi:10.3390/metabo9010010)
Supplement: Supplementary file 1 [file metabolites-09-00010-s001.pdf]

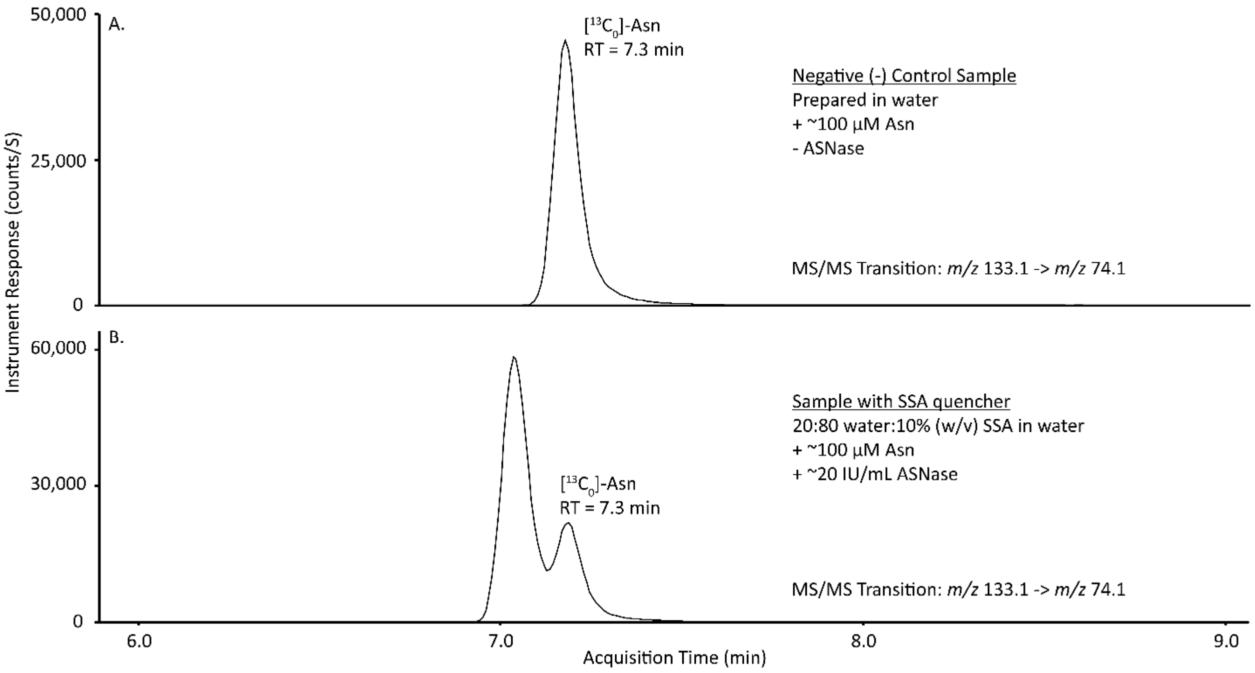

Figure S1. Sulfosalicylic Acid Exhibits an Effect on Asn Chromatography. Sulfosalicylic acid (SSA) has been previously reported as an effective quencher of ASNase activity in human clinical whole blood samples. We tested the quenching efficacy of a 8% (w/v) SSA solution on ASNase activity in a neat solution (20:80 water:10% (w/v) sulfosalicylic acid (SSA) in water) that contained an Asn concentration and ASNase activity of approximately 100 µM and 20 IU/mL, respectively, following the method described in Experimental Methods section. The presence of the SSA quencher in the neat sample did halt the ASNase-mediated conversion of Asn to Asp in the sample but proved to have deleterious effects on Asn chromatography (Figure S-2), so SSA was not chosen for use in the final method. Extracted-ion chromatograms (XICs) for the transitions monitored are shown for Asn for the following samples: A: a negative control sample prepared in water that contained approximately 100 µM of Asn and without ASNase; B: an SSA quenched sample that contained an Asn concentration and ASNase activity of approximately 100 µM and 20 IU/mL, respectively, in 20:80 water:10% (w/v) SSA in water. Although SSA was effective in quenching ASNase (no Asp formation was detected), the presence of the SSA quencher in the sample had an effect on the chromatographic retention of Asn for the HILIC-based chromatographic system described herein.

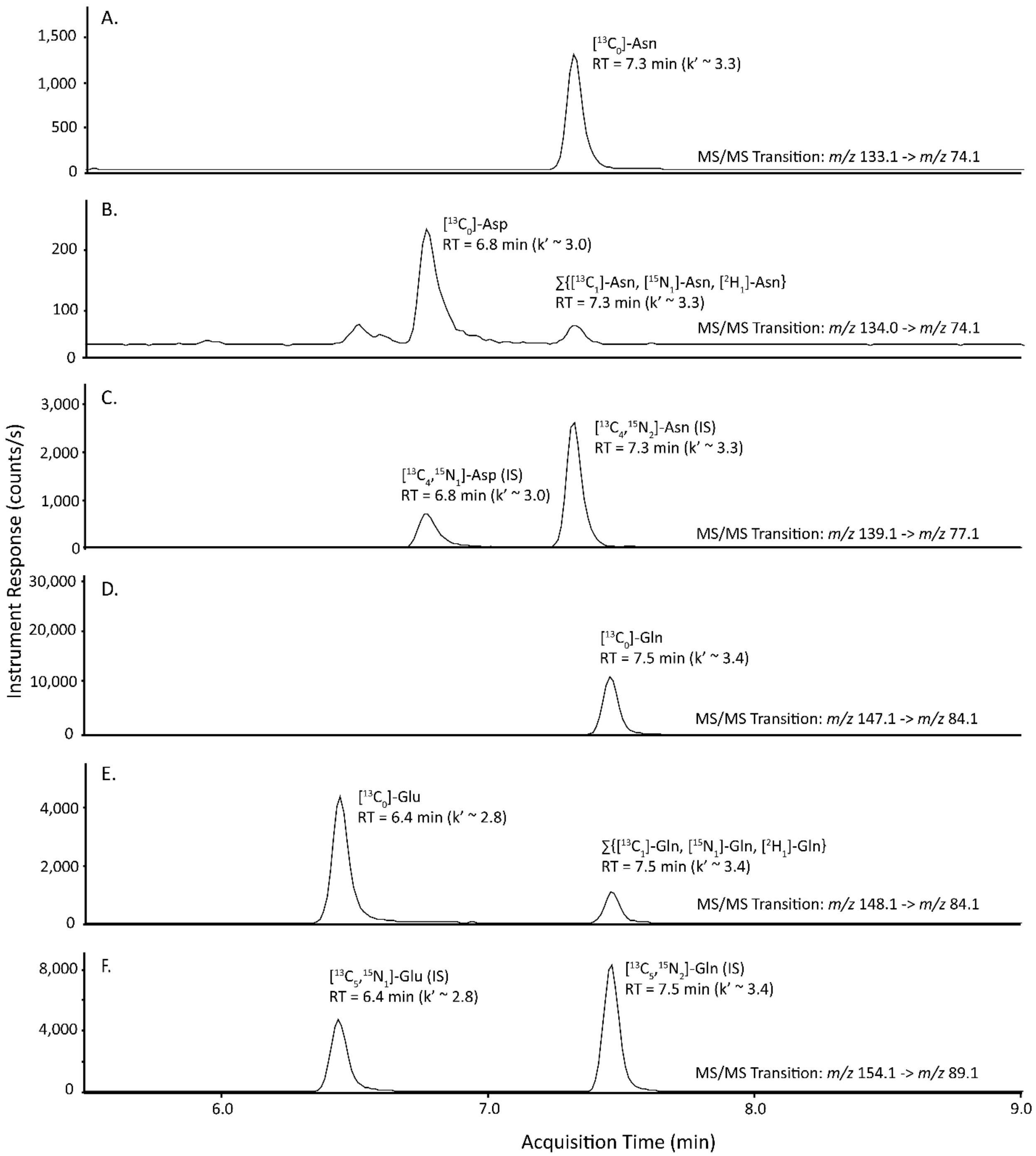

Figure S2. Extracted-ion chromatograms (XIC) for the transitions monitored. A: Asn; B: Asp (RT=6.8 min); C: [$^{13}C_4$,$^{15}N_2$]-Asn and [$^{13}C_4$,$^{15}N_1$]-Asp IS compounds; D: Gln; E: Glu (RT=6.4 min); F: [$^{13}C_5$,$^{15}N_2$]-Gln and [$^{13}C_5$,$^{15}N_1$]-Glu IS compounds. The chromatograms depicted here were acquired from a representative pre-treatment whole blood mouse study sample.

Table S1: Compiled Biological Reactions Involving Asparagine, Aspartic Acid, Glutamine, and Glutamic Acid

ASN

direction	substrate_or_product	gene
-->	AMP + an L-asparaginyl-[tRNAAsn] + diphosphate	NARS,NARS2
-->	L-aspartate + ammonium	ASRGL1,ASPG
<--	ATP + H2O + L-aspartate + L-glutamine	ASNS
<--	ATP + L-aspartate + ammonium	ASNS
<--	H2O + an L-asparaginyl-[tRNAAsn]	PTRH1,PTRH2
<--	N4-(&beta-N-acetyl-D-glucosaminyl)-L-asparagine + H2O	non-enzymatic

ASP

direction	substrate_or_product	gene
-->	5'-phosphoribosyl-4-(N-succinocarboxamide)-5-aminoimidazol	PAICS
-->	AMP + H+ + L-arginino-succinate + diphosphate	ASS1
-->	AMP + H+ + L-asparagine + L-glutamate + diphosphate	ASNS
-->	AMP + H+ + L-asparagine + diphosphate	ASNS
-->	AMP + an L-aspartyl-[tRNAasp] + diphosphate	DARS,DARS2
-->	GDP + H+ + adenylo-succinate + phosphate	ADSS,ADSSL1
-->	N-carbamoyl-L-aspartate + H+ + phosphate	CAD
--	H+ + NAD(P)H + ammonium + oxaloacetate	ASPDH
--	L-glutamate + oxaloacetate	GOT2
<-->	L-glutamate + oxaloacetate	GOT1,GOT1L1
<--	H2O + L-alanyl-L-aspartate	non-enzymatic
<--	H2O + L-asparagine	ASRGL1,ASPG
<--	H2O + a dipetide with an N-terminal L-aspartate	DPEP3
<--	H2O + a peptide with an N-terminal L-aspartate	DNPEP
<--	H2O + a protein	ASRGL1
<--	H2O + an N-acyl-L-aspartate	ASPA,ACY3
<--	N-acetyl-L-aspartate + H2O	non-enzymatic
<--	N4-(&beta-N-acetyl-D-glucosaminyl)-L-asparagine + H2O	AGA

GLN

direction	substrate_or_product	gene
-->	2-(formamido)-N1-(5-phospho-&beta-D-ribosyl)acetamidine +	PFAS
-->	5-phospho-&beta-D-ribosylamine + L-glutamate + diphosphate	PPAT
-->	ADP + CTP + H+ + L-glutamate + phosphate	CTPS1,CTPS2
-->	ADP + H+ + L-glutamate + an L-asparaginyl-[tRNAAsn] + phosph	QRSL1,PET112L
-->	ADP + H+ + L-glutamate + an L-glutaminyl-[tRNAGln] + phospha	PET112L,QRSL1
-->	ADP + H+ + L-glutamate + carbamoyl phosphate + phosphate	CAD
-->	AMP + GMP + H+ + L-glutamate + diphosphate	GMPS
-->	AMP + H+ + L-asparagine + L-glutamate + diphosphate	ASNS
-->	AMP + H+ + L-glutamate + NAD+ + diphosphate	NADSYN1
-->	AMP + an L-glutaminyl-[tRNAGln] + diphosphate	QARS
-->	L-glutamate + ammonium	GLS2,ASNS,GLS1

<-->	2-oxoglutaramate + L-methionine	CCBL1
<-->	2-oxoglutaramate + L-phenylalanine	CCBL1
<-->	D-glucosamine 6-phosphate + L-glutamate	GFPT1,GFPT2
<--	ATP + L-glutamate + ammonium	GLUL

GLU

direction	substrate_or_product	gene
-->	&gamma-L-glutamyl 5-phosphate + ADP	ALDH18A1
-->	&gamma-L-glutamyl-L-cysteine + ADP + H+ + phosphate	GCLM,GCLC
-->	(S)-1-pyrroline-5-carboxylate + H2O + NAD+	ALDH4A1
-->	2-oxoglutarate + 3-phospho-L-serine	PSAT1
-->	2-oxoglutarate + L-ornithine	non-enzymatic
-->	4-aminobutanoate + CO2	GAD1,GAD2,GLUL
-->	ADP + H+ + L-glutamine + phosphate	GLUL
-->	ADP + a 10-formyltetrahydrofolate + phosphate	FPGS
-->	ADP + a 5,10-methylenetetrahydrofolate + phosphate	non-enzymatic
-->	ADP + a tetrahydrofolate + phosphate	FPGS
-->	AMP + an L-glutamyl-[tRNAGln] + diphosphate	non-enzymatic
-->	AMP + an L-glutamyl-[tRNAGlu] + diphosphate	EPRS,EARS2
-->	H2O + L-glutamate-5-semialdehyde + NAD+	ALDH4A1
-->	N-acetyl-&alpha-L-aspartyl-L-glutamate + ADP + H+ + phosphat	RIMKLA
--	2-oxoglutarate + 4-phosphooxy-L-threonine	PSAT1
--	2-oxoglutarate + H+ + NADH + ammonium	non-enzymatic
--	2-oxoglutarate + L-aspartate	GOT2
--	N-acetyl-L-glutamate + H+ + coenzyme A	NAGS
<-->	&beta-D-fructofuranose 6-phosphate + L-glutamine	GFPT1,GFPT2
<-->	&beta-alanine + 2-oxoglutarate	ABAT
<-->	(S)-3-amino-2-methylpropanoate + 2-oxoglutarate	ABAT
<-->	2-oxoglutarate + 3-O-methyldopa	non-enzymatic
<-->	2-oxoglutarate + 3-hydroxy-L-kynurenine	CCBL2,AADAT
<-->	2-oxoglutarate + 3-sulfinoalanine	GOT1
<-->	2-oxoglutarate + L-alanine	GPT,GPT2
<-->	2-oxoglutarate + L-aspartate	GOT1,GOT1L1
<-->	2-oxoglutarate + L-cysteine	GOT1
<-->	2-oxoglutarate + L-dopa	non-enzymatic
<-->	2-oxoglutarate + L-kynurenine	CCBL2,CCBL1,GOT2,AADAT
<-->	2-oxoglutarate + L-lysine	non-enzymatic
<-->	2-oxoglutarate + L-tyrosine	TAT
<-->	2-oxoglutarate + erythro-4-hydroxy-L-glutamate	GOT2
<--	(glutathion-S-yl)-4-hydroxy-2-nonenal + H2O	non-enzymatic
<--	2-oxoglutarate + 4-aminobutanoate	ABAT
<--	2-oxoglutarate + L-2-aminoadipate	AADAT
<--	2-oxoglutarate + L-isoleucine	BCAT1,BCAT2
<--	2-oxoglutarate + L-leucine	BCAT1,BCAT2
<--	2-oxoglutarate + L-valine	BCAT1,BCAT2
<--	5-oxo-L-proline + ATP + H2O	OPLAH

<--	5-phospho-&alpha-D-ribose 1-diphosphate + H2O + L-glutamin	PPAT
<--	ATP + H2O + L-aspartate + L-glutamine	ASNS
<--	ATP + H2O + L-glutamine + UTP	CTPS1,CTPS2
<--	ATP + H2O + L-glutamine + XMP	GMPS
<--	ATP + H2O + L-glutamine + an L-aspartyl-[tRNAAsn]	QRSL1,PET112L
<--	ATP + H2O + L-glutamine + an L-glutamyl-[tRNAGln]	PET112L,QRSL1
<--	ATP + H2O + L-glutamine + hydrogencarbonate	CAD
<--	ATP + H2O + L-glutamine + nicotinate adenine dinucleotide	NADSYN1
<--	H2O + L-glutamine	GLS2,ASNS,GLS1
<--	H2O + L-saccharopine + NAD+	AASS
<--	H2O + a glutathione-S-conjugate	GGT5,GGT1
<--	H2O + a peptide	FOLH1,NAALADL1,NAALAD2,FOLH1B
<--	H2O + a tetrahydrofolate	non-enzymatic
<--	H2O + an N-terminal L-glutamyl-[protein]	ENPEP
<--	H2O + glutathione	GGT1,GGT2,GGT5
<--	H2O + leukotriene-C4	GGT5,GGT1
<--	N-formimino-L-glutamate + a tetrahydrofolate	FTCD
<--	N2-formyl-N1-(5-phospho-&beta-D-ribosyl)glycinamide + ATP +	PFAS
